# Direct evidence of fiber-protein-directed hemagglutination by canine adenoviruses

**DOI:** 10.1007/s00705-023-05718-5

**Published:** 2023-02-16

**Authors:** Hiromichi Matsugo, Haruhiko Kamiki, Hiroho Ishida, Tomoya Kobayashi-Kitamura, Akiko Takenaka-Uema, Shin Murakami, Taisuke Horimoto

**Affiliations:** Laboratory of Veterinary Microbiology, Graduate School of Agricultural and Life Sciences, The University of Tokyo, Tokyo, Japan

**Keywords:** Canine adenovirus, Fiber, Hemagglutination, Receptor

## Abstract

Canine adenoviruses (CAdVs) are divided into two serotypes, CAdV1 and CAdV2, whose members mainly cause infectious hepatitis and laryngotracheitis, respectively, in canids. To gain insight into the molecular basis of viral hemagglutination, we constructed chimeric viruses whose fiber proteins or their knob domains, which play a role in viral attachment to cells, were swapped among CAdV1, CAdV2, and bat adenovirus via reverse genetics. The results revealed that, in each case, viral hemagglutination was specifically mediated by the fiber protein or knob domain, providing direct evidence for fiber-protein-directed receptor-binding characteristics of CAdVs.

Canine adenoviruses (CAdVs) are classified as members of the species *Canine mastadenovirus A*, genus *Mastadenovirus*, family *Adenoviridae.* They are divided into two serotypes: CAdV1 and CAdV2. CAdV1 has a broad host range and causes infectious hepatitis and encephalitis in dogs, bears, red foxes, and wolves [1–5]. CAdV2 causes infectious laryngotracheitis in dogs [1, 6], while attenuated CAdV2, which cross-reacts serologically with CAdV1, is widely used as a vaccine. The molecular basis of the pathogenic differences between CAdV1 and CAdV2 cannot be deduced from genome sequence comparisons [7–9]. Previous studies have indicated that ferrets and guinea pigs are susceptible to CAdV1 infection [10, 11], providing an animal model for laboratory-based pathogenic studies of CAdVs.

The AdV capsid consists of three main structural proteins: hexon, penton base, and fiber. Fiber proteins facilitate viral entry into cells by binding to cellular receptors. One of these cellular receptors is associated, to some extent, with tissue tropism in human AdVs [12, 13]. This suggests a possible mechanistic explanation for differences in the pathogenicity of the CAdV serotypes in which clinical outcomes may be determined by differences in receptor-mediated tissue tropism.

Interestingly, CAdV1 and CAdV2 exhibit differences in their hemagglutination (HA) activity. CAdV1 agglutinates erythrocytes of a wide range of animal species, including humans, rats, guinea pigs, and birds, whereas CAdV2 agglutinates a narrower range of animal erythrocytes, including only those from humans and rats [1, 14, 15]. Notably, neither virus robustly agglutinates dog erythrocytes, suggesting the absence of a direct association between the HA profile and viral host specificity. Nonetheless, these findings imply that the receptors for these viruses on erythrocytes vary. Previous studies have shown that both CAdV1 and CAdV2 can use the coxsackievirus and adenovirus receptor (CAR), but they may also use unknown alternative receptors [16, 17]. CAR is found on the basolateral surface, but not the apical surface, of intact polarized airway epithelia [18, 19]. This indicates that CAR may not be the primary receptor for CAdVs *in vivo* and that other unknown receptors may determine viral tissue tropism. These observations also suggest that differences in the viral fiber proteins could result in differences in tissue tropism, which in turn could be responsible for some of the differences in the pathogenic properties between CAdV1 and CAdV2. In this study, to gain insight into the molecular basis of receptor binding of CAdVs, we analyzed the HA activity of chimeric viruses whose fiber proteins were swapped between the two viruses.

We generated fiber-protein-swapped chimeric mutants of CAdVs using a bacterial artificial chromosome (BAC)-based reverse genetics system [20]. Two pSMART BAC-based vectors (Lucigen, Middleton, WI, USA) were constructed. These include pBAC-CAdV1, creating a DNA insert from CAdV1 strain D43 (GenBank accession no. LC557010) and pBAC-CAdV2, containing a DNA insert from CAdV2 strain A2 (GenBank accession no. LC557011) [20]. These constructs were then used as templates to construct recombinant BAC plasmids to generate mutant viruses. Briefly, galactokinase (GalK)-kanamycin resistance gene (Kn) cassettes were amplified from pGalK-Kn via PCR using the primers CAdV1-fiber-GalK-Kn F/R and CAdV2-fiber-GalK-Kn F/R, sequences of which are available upon request. SW102 cells containing pBAC-CAdV1 or pBAC-CAdV2 were heat-shocked to induce red recombinase expression and electroporated with their respective PCR products using a MicroPulser (Bio-Rad, Hercules, CA, USA). The recovered cells were plated on Luria-Bertani (LB) medium containing chloramphenicol (CP) and kanamycin (KM), and bacterial colonies were passaged on MacConkey agar plates containing CP and d-galactose. The red bacterial colonies on MacConkey plates, which were GalK positive, were subsequently incubated in LB medium containing CP and KM. After incubation, red recombinase was induced via heat shock. The CAdV1 or CAdV2 fiber genes were amplified by PCR using the primers CAdV2-CAdV1-fiber F/R or CAdV1-CAdV2-fiber F/R. The cells were transformed with purified PCR products to replace the GalK-Kn cassettes. After incubation, the cells were washed with M9 medium and plated onto M63 minimal medium plates containing glycerol, leucine, biotin, 2-deoxy-galactose, and CP. The bacterial colonies were then screened, and isolated positive colonies were selected. Chimeric BAC constructs were then made in which the CAdV1 and CAdV2 fiber genes were swapped between pBAC-CAdV1 and pBAC-CAdV2 (namely, pBAC-CAdV1/2-fiber and pBAC-CAdV2/1-fiber). Linearized viral genomic DNA was released from each BAC vector using a restriction enzyme and introduced by transfection into Madin-Darby canine kidney (MDCK) cells using polyethyleneimine (Polysciences, Warrington, PA, USA). At 5–6 days post-transfection, the medium and cells were frozen and thawed three times, and the supernatants obtained after centrifugation of the cell cultures were collected and used to infect MDCK cells. The culture supernatants were also collected and stored at -80°C when 50–80% of the cells showed a cytopathic effect. The CAdV1 mutant containing the CAdV2 fiber protein was named CAdV1/2-fiber, and the CAdV2 mutant containing the CAdV1 fiber protein was named CAdV2/1-fiber (Fig. 1A). The sequences adjacent to the genetically modified regions of the recombinant viruses were confirmed before performing subsequent experiments. Recombinant wild-type CAdV1 (CAdV1-rWT) and CAdV2 (CAdV2-rWT), generated using BAC-based reverse genetics [20], were also used for comparative analysis. In addition, we constructed fiber knob domain-swapped chimeric mutants of CAdV2 and bat adenovirus (BtAdV) employing pSMART BAC vectors. pBAC-CAdV2-Toronto contained DNA from the CAdV2 Toronto strain (GenBank accession no. U77082), and pBAC-BtAdV-Mm32 contained DNA from the BtAdV Mm32 strain (GenBank accession no. LC385828) [16, 21]. The CAdV2 mutant containing the BtAdV fiber knob domain and the BtAdV mutant containing the CAdV2-Toronto fiber knob domain were named CAdV2-To/BtAdV-knob and BtAdV/CAdV2-To-knob, respectively (Fig. 1B). Recombinant wild-type CAdV2 Toronto strain (CAdV2-To-rWT) and BtAdV (BtAdV-rWT) were also generated.

**Fig. 1:**
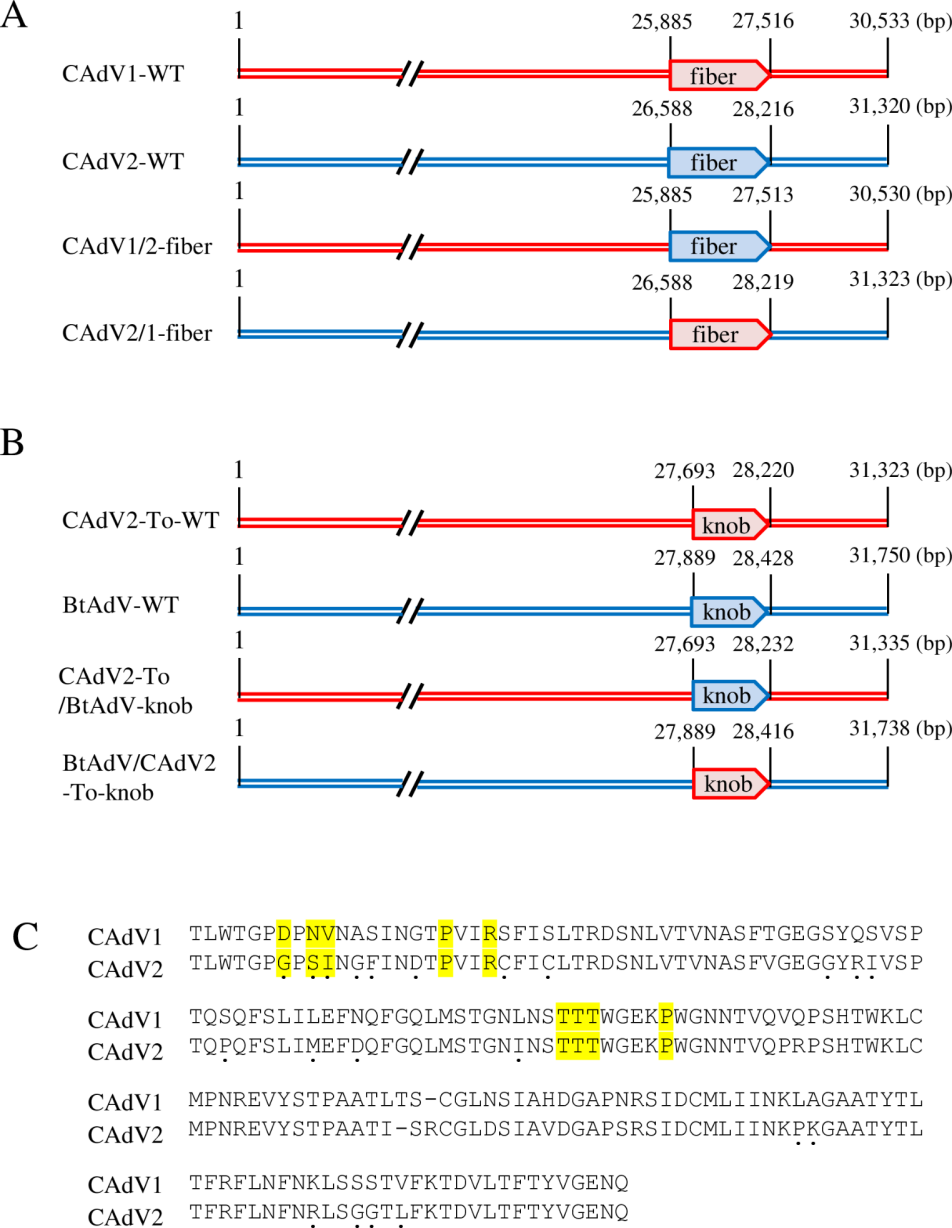
Characteristics of the fiber-protein-swapped canine adenoviruses (CAdVs) and the fiber knob domains of fiber-protein-swapped CAdV2 and bat adenovirus (BtAdV). (A and B) Genome structure of the chimeric viruses (A) whose fiber proteins were swapped between CAdV1 and CAdV2 (A2 strain) and (B) whose fiber knob domains were swapped between CAdV2 (Toronto strain) and BtAdV. (C) Amino acid sequence alignment of the fiber knob domains of the fiber proteins. Residues that differ between CAdV1 and CAdV2 (A2 strain) are indicated by a dot (•). Residues that play an important role in the interaction with CAR are highlighted in yellow.

To examine viral HA activity, we serially diluted viruses (1 × 10^9^ PFU/mL for CAdV1-rWT, CAdV2-rWT, CAdV1/2-fiber, and CAdV2/1-fiber or 1 × 10^8^ PFU/mL for CAdV2-To-rWT, BtAdV-rWT, CAdV2-To/BtAdV-knob, and BtAdV/CAdV2-To-knob) twofold and incubated them with a 0.7% suspension of turkey, guinea pig, or human erythrocytes in phosphate-buffered saline on ice for 1 h. The HA titer was defined as the highest dilution of the virus that completely aggregated erythrocytes. Turkey and guinea pig erythrocytes were purchased from Japan Bio Serum (Hiroshima, Japan). Human erythrocytes were obtained from a healthy individual, with ethical permission from the Research Ethics Committee of the Graduate School of Agricultural and Life Sciences at the University of Tokyo (approval number 20–258). CAdV1-rWT showed HA activity with erythrocytes from all three animals, whereas CAdV2-rWT only showed HA with human erythrocytes (Table 1). These results confirmed those of a previous study showing a qualitative difference in HA properties between CAdV1 and CAdV2. However, turkey and human type A erythrocytes were used for our assay instead of the fowl/goose and human type O erythrocytes, respectively, that were used previously [14]. Adenoviral HA activity is dependent upon the presence of available receptors, and fiber proteins have affinity for such receptors on erythrocytes [22]. Therefore, the different HA titers for each erythrocyte type with CAdV1-rWT and the HA profile of each type of erythrocyte with CAdV2-rWT strongly suggest that the receptor molecules responsible for HA were qualitatively or quantitatively different among the erythrocyte types, especially those between turkeys or guinea pigs and humans. Previous studies have shown that CAR on erythrocytes is responsible for the HA activity of CAdV2 [22] and that CAdV1 uses CAR as a receptor for infection [16], suggesting that CAR is also responsible for the HA activity of CAdV1. It has also been reported that CAR is expressed on human and rat erythrocytes, but not on those of dogs, mice, rabbits, or non-human primates [22]. However, the presence of CAR or CAR homologs on guinea pig or turkey erythrocytes has not been investigated. Further studies are needed to clarify the role of receptor molecules on erythrocytes of different species in the HA activity of CAdV1 and CAdV2.

**Table 1:** Hemagglutination activity of the recombinant CAdVs or BtAdV

Virus	Source of erythrocytes								
	Turkey			Guinea pig			Human		
	Exp 1	Exp 2	Exp 3	Exp 1	Exp 2	Exp 3	Exp 1	Exp 2	Exp 3
CAdV1-rWT	32	32	16	64	64	64	256	256	256
CAdV2-rWT	< 2	< 2	< 2	< 2	< 2	< 2	16384	16384	16384
CAdV1/2-fiber	< 2	< 2	< 2	< 2	< 2	< 2	512	256	256
CAdV2/1-fiber	512	256	256	512	512	512	8192	8192	8192
CAdV2-To-rWT	ND	ND	ND	ND	ND	ND	64	64	64
BtAdV-rWT	ND	ND	ND	ND	ND	ND	< 2	< 2	< 2
CAdV2-To/BtAdV-knob	ND	ND	ND	ND	ND	ND	< 2	< 2	< 2
BtAdV/CAdV2-To-knob	ND	ND	ND	ND	ND	ND	64	64	64

Each assay was repeated independently three times (experiments 1, 2, and 3). The titers are expressed as the reciprocal of the highest dilution of the virus that showed complete HA of each erythrocyte type. ND, not determined

Next, we examined the HA activity of the chimeric viruses. CAdV1/2-fiber showed HA activity only with human erythrocytes, as did CAdV2-rWT. In contrast, CAdV2/1-fiber showed HA with all three erythrocyte types, as did CAdV1-rWT (Table 1). CAdV2-To/BtAdV-knob and BtAdV-rWT exhibited no HA activity with human erythrocytes, unlike CAdV2-To-rWT. However, unlike BtAdV-rWT, BtAdV/CAdV2-To-knob showed a level of HA activity with human erythrocytes that was comparable to that of CAdV2-To-rWT (Table 1). Collectively, these findings suggest that the swapped fiber proteins and their knob domains determine the HA specificity of the chimeric viruses. Therefore, the fiber proteins alone might determine the receptor-binding properties of CAdVs. These results indicate that the CAdV1 fiber protein, but not the CAdV2 fiber protein, can attach to receptors on turkey and guinea pig erythrocytes and that the affinity of the CAdV1 fiber protein for these receptors is much higher than that of CAdV2. In contrast, both CAdV1 and CAdV2 fiber proteins were able to attach to the receptors on human erythrocytes, suggesting that different surface receptor molecules might be used on human erythrocytes than on turkey and guinea pig erythrocytes.

The amino acid sequences of the whole fiber proteins and the fiber knob domains that directly bind to the receptors (amino acid residues 365–543 for CAdV1 and 36–542 for CAdV2) are 80% and 83% identical, respectively, between CAdV1 and CAdV2. These amino acids, which play an important role in the interaction between the fiber knob domain and CAR [23], are not completely conserved between CAdV1 and CAdV2 (Fig. 1C), suggesting that the affinity of the fiber proteins for CAR might be different between the two viruses. In addition, it is likely that binding of CAdV1 to turkey or guinea pig erythrocytes is mediated by nonspecific electrostatic interactions between negatively charged molecules, such as sialic acids, on erythrocytes and the CAdV1 fiber knob domain. Research has demonstrated the presence of such interactions between sialic acids and positively charged adenovirus fiber knob domains, including that of CAdV2 [22, 24]. However, the predicted isoelectric point (6.6) of the fiber knob domain of CAdV1 is lower than that of CAdV2 (8.4), which showed no HA activity with turkey or guinea pig erythrocytes. Therefore, the ability of CAdV1 to agglutinate turkey or guinea pig erythrocytes may not be due to nonspecific electrostatic interactions.

The HA titers of the CAdV2/1-fiber were 8- to 32-fold higher than those of CAdV1-rWT for all erythrocyte types, even though the same number of plaque-forming units (PFU) of each virus was used for the assay, suggesting different ratios of virus particles per PFU between the two viruses. In contrast, the HA titers for the CAdV1/2-fiber were 32-to 64-fold lower than those of CAdV2-rWT in human erythrocytes. Although we could not determine the number of virus particles per PFU, these ratios were negatively correlated with growth efficiency [25]. CAdV2 growth was less efficient than that of CAdV1, suggesting that CAdV2 has a higher ratio of viral particles per PFU. In addition, the artificial generation of recombinant viruses via reverse genetics may produce non-infectious viral particles, resulting in different ratios. Alternatively, other capsid proteins, such as hexon and penton bases, may affect the viral structure in the chimeric forms, leading to differences in HA titers.

We also examined the growth properties of the chimeric viruses in MDCK and canine fibrosarcoma A72 cells [26]. Both chimeric viruses, CAdV1/2-fiber and CAdV2/1-fiber, replicated in a similar manner to the parental CAdV1-rWT and CAdV2-rWT strains in both canine cell lines (Fig. 2). Moreover, the cytotoxicity and plaque morphology of the mutants were similar to those of parental viruses (data not shown). These data indicated that swapping the fiber proteins did not affect the growth of CAdVs in these two dog-derived cell lines, demonstrating that the CAdV1 and CAdV2 fiber proteins are functionally compatible with viral growth in these cell lines. Additional tests using other dog-organ-derived cell cultures, including organoids, and evaluation of the CAR content in each cell line are needed to investigate the receptor-mediated tissue tropism of CAdVs.

**Fig. 2:**
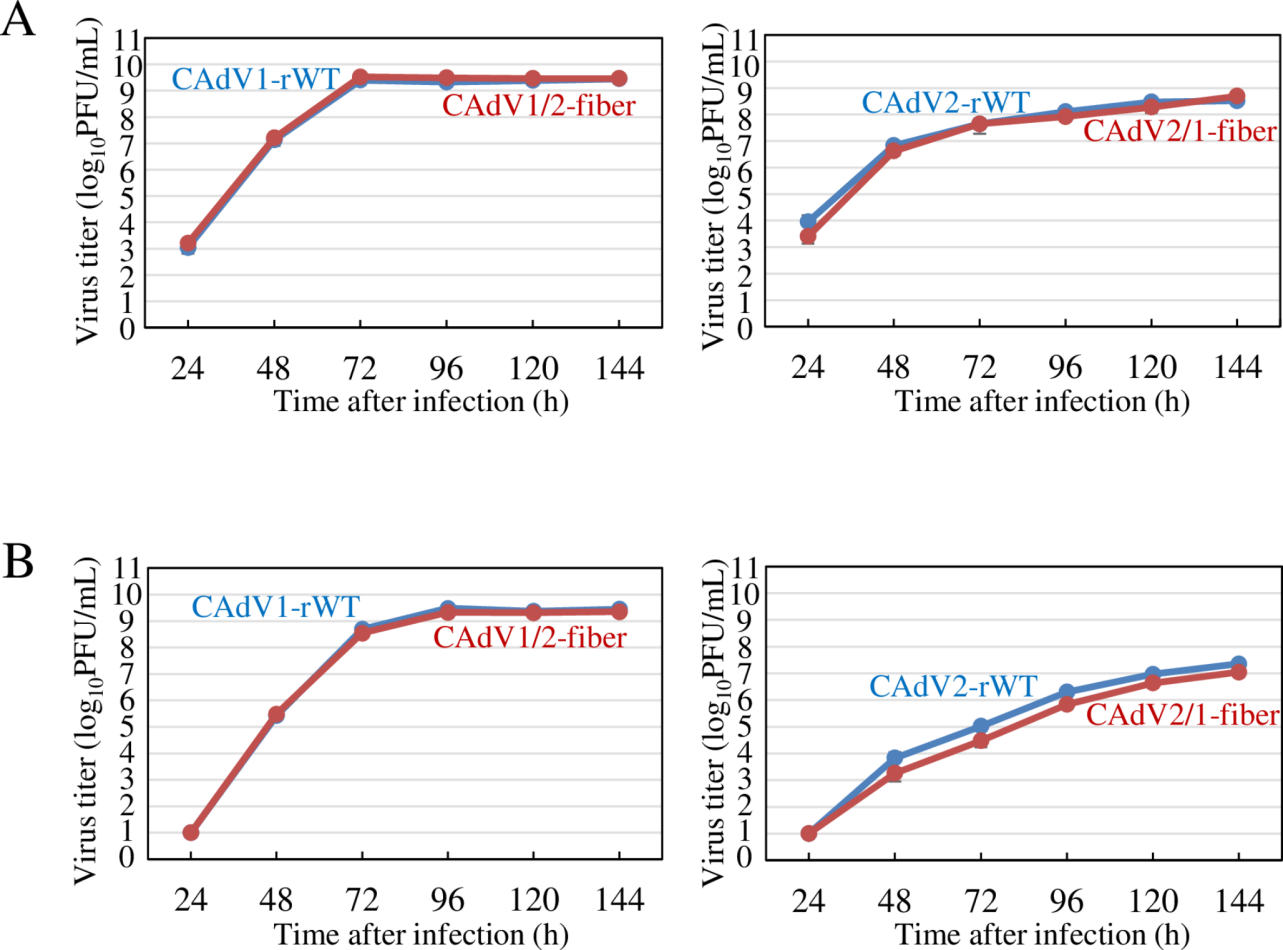
Growth kinetics of fiber-protein-swapped CAdVs. MDCK (A) or A72 (B) cells were infected with CAdV1-rWT, CAdV1/2-fiber, CAdV2-rWT, or CAdV2/1-fiber at a multiplicity of infection of 0.01. The culture supernatants were collected at the indicated time points. Viral titers were determined using a plaque assay and are reported as the mean titer with standard deviation for three independent experiments.

In conclusion, we have provided direct evidence of fiber-protein-directed binding of CAdVs to erythrocyte receptors. This finding may provide clues to the receptor-regulated determinants of CAdV pathogenesis.
